# Enhancing Caudal Analgesia in Children: A Narrative Review of Clonidine and Dexmedetomidine as Adjuvants

**DOI:** 10.7759/cureus.99500

**Published:** 2025-12-17

**Authors:** Anuradha Vaswani, Khushboo Bairwa, Richa Kewalramani, Jaiprakash Gangani, Vidya Mohan

**Affiliations:** 1 Department of Anesthesiology and Critical Care, All India Institute of Medical Sciences, Jodhpur, IND; 2 Department of Anesthesiology, Kshetrapal Multispeciality Hospital and Research Center, Ajmer, IND; 3 Department of General Medicine, JIET Medical College and Hospital, Jodhpur, IND

**Keywords:** analgesia, caudal block, clonidine, dexmedetomidine, pediatrics, regional anesthesia, sedation, α2-adrenergic agonists

## Abstract

Caudal epidural analgesia is a common method of pediatric anesthesia for infraumbilical and lower limb operations. Despite being useful, the limited duration of action of local anesthetics by themselves necessitates the addition of adjuvants to prolong postoperative pain relief. Among them, clonidine and dexmedetomidine, both α2-adrenergic agonists, have become popular because of their analgesic-sedative effects in combination. This review discusses the pharmacology, clinical efficacy, safety, and comparison of dexmedetomidine and clonidine as adjuvants for pediatric caudal blocks. Randomized controlled trials, meta-analyses, and systematic reviews were used in combination to compare their roles. The significant outcomes are quality and duration of analgesia, hemodynamic stability, sedation, and side effects. Both drugs significantly prolong the duration of caudal analgesia, reduce postoperative opioid consumption, and exhibit good safety profiles. Dexmedetomidine gives better pain relief and enhanced sedation due to its greater α2-receptor selectivity but also poses a higher risk for bradycardia and hypotension. Clonidine, though less potent, shows consistent effectiveness, is cost-efficient, and has a safer hemodynamic profile, making it especially suitable for outpatient procedures. Clonidine and dexmedetomidine are effective adjuvants for enhancing pediatric caudal analgesia. While dexmedetomidine is better for longer and more painful surgeries, clonidine offers a balanced option when quick recovery and stable blood pressure are priorities. Future large-scale studies and long-term neurodevelopmental safety research are necessary to optimize their clinical use.

## Introduction and background

Pediatric anesthesia has advanced considerably in the last few decades, yet perioperative pain management in children remains a significant clinical challenge. Caudal epidural analgesia is among the most widely practiced regional anesthetic techniques in pediatric patients, particularly for infraumbilical and lower limb surgeries [1-3]. Its popularity stems from the relative simplicity of the procedure, predictable spread of local anesthetic, and effectiveness in providing intraoperative as well as postoperative analgesia. Despite these benefits, the only significant drawback of caudal anesthesia is the comparably short duration of effect when done with local anesthetics only. Surgical children usually need prolonged relief of pain, and the requirement for multiple systemic analgesics raises the risk of drug side effects and prolongs recovery [4-7].

In an effort to surpass these limitations, various adjuvants have been experimented with in combination with local anesthetics in caudal analgesia. The purpose of these adjuvants is to enhance the depth and duration of analgesia, reduce the overall dose of the local anesthetic administered, and reduce systemic requirement for analgesics [8,9]. Among the pharmacological agents studied, α2-adrenergic agonists clonidine and dexmedetomidine have gained significant interest in the clinic due to their combined roles as analgesics and sedatives [10-13].

The older of the two, clonidine, is a weak partial α2-adrenergic agonist that has central and peripheral actions that contribute to its analgesic effects. Dexmedetomidine, on the other hand, is a highly selective α2-adrenergic agonist with enhanced receptor affinity, which produces stronger sedative and analgesic effects. Both drugs have been utilized in numerous clinical uses, including intensive care unit sedation, regional anesthesia, and perioperative pain management [1,2,5,6]. Their use in children's caudal anesthesia holds particular promise because these drugs can potentially extend the duration of analgesia with minimal impact on motor block or systemic side effects [14,15].

Existing clinical reviews and trials confirm that clonidine and dexmedetomidine can prolong the duration of caudal blocks, improve postoperative pain relief, and decrease the need for rescue analgesics [8,9,11]. Furthermore, these medications possess an acceptable safety profile when given in appropriate doses, with issues like bradycardia, hypotension, and sedation still useful considerations in children [12,13]. An understanding of the pharmacology, clinical information, and comparative efficacy of these agents is therefore relevant to anesthesiologists who want to maximize perioperative outcomes in children.

The purpose of this narrative review is to synthesize current evidence concerning the use of clonidine and dexmedetomidine as adjuvants in children's caudal analgesia. A narrative review design was deliberately chosen over a systematic or quantitative meta-analytic approach because of the existing heterogeneity and limited standardization across published studies on pediatric caudal adjuvants. Existing research varies considerably in terms of patient demographics, surgical types, dosing protocols, adjuvant combinations, and outcome assessment tools. Such variability prevents meaningful data pooling or meta-analytic synthesis without introducing substantial bias. Moreover, several key studies in this domain are observational, single-center, or exploratory in nature, making them unsuitable for inclusion in a formal meta-analysis. The narrative format, therefore, provides the flexibility to integrate findings from randomized controlled trials, meta-analyses, and pharmacological studies while maintaining a critical interpretive framework. This approach facilitates a comprehensive and context-rich understanding of clinical efficacy, safety, and applicability, aligning with the review’s objective to inform practical anesthetic decision-making rather than generate pooled statistical estimates.

The subsequent sections discuss the pharmacological rationale, clinical efficacy, safety, and relative effectiveness of clonidine and dexmedetomidine, along with practical considerations for their administration. The review also identifies gaps in the current literature and suggests future research directions in pediatric analgesia. By doing so, it provides anesthesiologists and pediatric clinicians with an integrated overview of two of the most widely researched and clinically relevant adjuvants used in caudal anesthesia. The characteristics that define adjuvants, their advantages and disadvantages, and the rationale for their use in caudal analgesia are enumerated in Table 1 and form the foundation for understanding the clinical application of clonidine and dexmedetomidine in pediatric practice.

**Table 1:** Key characteristics of caudal analgesia in children.

Parameter	Details	References
Primary indications	Infraumbilical, lower limb, urological, and abdominal surgeries	[1-3]
Advantages	Simple to perform, reliable analgesia, reduced systemic opioid use	[4-7]
Limitations (without adjuvants)	Short duration of action (4–6 hours), need for repeat dosing, systemic analgesic requirement	[8,9]
Commonly used local anesthetics	Bupivacaine, ropivacaine, levobupivacaine	[10-12]
Rationale for adjuvants	Prolong analgesia, improve block quality, minimize systemic drug load	[13-15]

In summary, caudal anesthesia is a valuable resource for pediatric anesthesiologists, but when it is combined with adjuvants such as clonidine and dexmedetomidine, its effectiveness can be significantly increased. During the following review, both of these medications will be fully characterized, including pharmacologic effects, trial data, safety profiles, and relative advantages. This review will provide a rational clinical decision-making context and highlight areas for further research in pediatric regional anesthesia.

## Review

Caudal analgesia in children

Caudal epidural analgesia has been a cornerstone of pediatric regional anesthesia, particularly for infraumbilical and lower extremity surgeries. Caudal block has been reported to be performed in more than 50% of regional anesthesia procedures in children because of its variability and safety profile [16,17]. The following discussion provides an overview of anatomy, technique, pharmacology, and clinical limitations of caudal analgesia in children.

Definition and clinical significance

Caudal epidural analgesia is a one-shot local anesthetic injection into the caudal epidural space through the sacral hiatus. Caudal epidural analgesia is useful for analgesia in below-umbilical surgeries like herniotomies, circumcisions, orchidopexies, and lower limb orthopedic operations [18,19]. In comparison to systemic opioid administration, caudal blocks reduce intraoperative anesthetic needs, allow smoother emergence from anesthesia, and reduce postoperative pain scores [20-22].

Anatomy and technique of caudal block

The caudal epidural space is reached through the sacral hiatus, which is flanked by the sacral cornua and overlain by the sacrococcygeal ligament. After identifying anatomical landmarks, a short bevel needle is advanced into the caudal canal, and the correct placement is confirmed by lack of resistance and negative aspiration. Ultrasound guidance, though not always necessary, has gained popularity as it improves accuracy, reduces the risk of vascular puncture, and allows visualization of local anesthetic spread [23-25]. Once the needle is placed, a single dose of local anesthetic, commonly bupivacaine, ropivacaine, or levobupivacaine, is administered. The block produces segmental analgesia extending from the sacral to thoracolumbar dermatomes, depending on volume and concentration used [18,19].

Commonly used local anesthetics

In pediatric caudal anesthesia, the primary local anesthetics used are bupivacaine, ropivacaine, and levobupivacaine. Each of these drugs provides distinct advantages and limitations regarding efficacy and safety profiles. Bupivacaine is a long-acting local anesthetic that provides a reliable sensory block, making it effective for managing pain during pediatric surgeries [26]. However, its use is limited by the risk of cardiotoxicity at higher plasma concentrations, which becomes more pronounced when higher doses or repeated administrations are required. Ropivacaine, although similar in duration to bupivacaine, is often preferred in pediatric anesthesia due to its reduced cardiotoxicity [27]. This makes it a safer option, particularly for younger patients, as it carries a lower risk of adverse cardiovascular effects. Levobupivacaine, the S-enantiomer of bupivacaine, offers an alternative with similar efficacy in terms of sensory block but with an improved safety profile, particularly regarding reduced cardiovascular toxicity [28-30]. This makes levobupivacaine a valuable option in pediatric caudal anesthesia, balancing effective pain management with a safer side-effect profile.

Typically, the analgesic effects of these drugs last between three and six hours [31]. In cases of prolonged surgeries or when extended postoperative pain relief is required, repeated dosing or the use of a caudal catheter for continuous infusion may be necessary. However, this approach increases the risk of infection and complications related to catheter placement, necessitating careful management to ensure patient safety.

Challenges and limitations

Despite its effectiveness, caudal analgesia without adjuvants has several limitations. Analgesia typically lasts for only a few hours, necessitating the use of systemic analgesics postoperatively. The spread of the block can also vary, as factors such as age, injection volume, and technique influence the extent and duration, often leading to inconsistent results [32]. Though rare, complications like dural puncture, local anesthetic systemic toxicity, infection, and motor block may occur, which can delay mobilization [29]. These limitations emphasize the importance of adjuvants, such as clonidine and dexmedetomidine, to improve the quality and duration of analgesia [33]. A comparative overview of bupivacaine, ropivacaine, and levobupivacaine is provided in Table 2, which highlights their respective advantages, limitations, and clinical considerations.

**Table 2:** Comparison of common local anesthetics in pediatric caudal analgesia.

Local anesthetic	Duration of analgesia	Advantages	Limitations	References
Bupivacaine	3–6 hours	Reliable sensory block, widely studied	Cardiotoxicity at high levels	[20-22]
Ropivacaine	3–6 hours	Lower cardiotoxicity than bupivacaine, good motor-sensory differentiation	Slightly less potent	[20-22]
Levobupivacaine	3–6 hours	Safer stereoisomer of bupivacaine, similar efficacy	Limited availability in some regions	[20-22]

Rationale for adjuvants

The short duration of single-shot caudal blocks has led to sustained interest in identifying safe and effective adjuvants. An ideal adjuvant should prolong the duration of analgesia without significantly increasing motor block, provide additional benefits such as sedation or hemodynamic stability, and be safe, particularly in children, with minimal systemic absorption and side effects [34]. Opioids, ketamine, and neostigmine have all been explored as adjuvants, but their use is limited due to concerns about side effects like respiratory depression, neurotoxicity, and inconsistent efficacy [35-37]. In contrast, α2-adrenergic agonists like clonidine and dexmedetomidine have demonstrated a favorable balance of efficacy and safety, making them the most studied adjuvants in this setting [31].

Agents such as opioids, ketamine, and neostigmine have been explored, but concerns about respiratory depression, neurotoxicity, and inconsistent efficacy have limited their widespread use [32]. In contrast, α2-adrenergic agonists like clonidine and dexmedetomidine have demonstrated a favorable balance of efficacy and safety, making them the most studied adjuvants in this setting [38-41].

Pharmacological basis of adjuvants in caudal block

Adjuvants are used in pediatric caudal anesthesia to address the limitations of local anesthetics alone. While agents like bupivacaine, ropivacaine, and levobupivacaine remain the foundation of caudal blocks, their short duration of action (typically three to six hours) makes them inadequate for surgeries requiring prolonged postoperative analgesia [42]. Adjuvants are therefore introduced to prolong block duration, enhance analgesic quality, and reduce the need for systemic opioids or repeat dosing [43,44]. Among these, α2-adrenergic agonists, specifically clonidine and dexmedetomidine, have demonstrated remarkable clinical utility due to their unique pharmacological profiles [45].

Rationale for adding adjuvants

The addition of adjuvants to caudal anesthesia aims to achieve several therapeutic goals. By potentiating the effects of local anesthetics at the spinal level, adjuvants extend the duration of sensory blockade without significantly increasing motor block [46,47]. Adjuvants also allow for the use of lower doses of local anesthetics, which decreases the risk of toxicity while maintaining analgesic efficacy [48]. Combining agents with different mechanisms of action provides superior pain relief compared to monotherapy, and a prolonged block reduces the need for opioids or NSAIDs postoperatively, lowering the risks of respiratory depression, nausea, and gastrointestinal complications [34].

Desired characteristics of an ideal adjuvant

An ideal adjuvant in pediatric caudal anesthesia should be able to prolong sensory blockade without prolonging motor block, possess minimal systemic absorption and low central nervous system toxicity, and maintain hemodynamic stability in children [49]. Additionally, it should be easy to administer as a single bolus with predictable pharmacokinetics and demonstrate safety across various age groups and surgical settings [50]. While opioids, ketamine, and neostigmine have been trialed as adjuvants, none fully meet these requirements [51]. Their side-effect profiles, such as pruritus and respiratory depression with opioids, neurotoxicity concerns with ketamine, and inconsistent results with neostigmine, make them less suitable for routine use in pediatric patients [52].

Mechanisms of action of α2-adrenergic agonists

Both clonidine and dexmedetomidine act via α2-adrenergic receptors located in the spinal cord, brainstem, and peripheral nerves. At the spinal level, they inhibit substance P and glutamate release, reducing nociceptive transmission [36]. Supraspinally, they suppress locus coeruleus activity, producing sedation and anxiolysis [53]. Peripherally, they reduce norepinephrine release and dampen pain signaling. Together, these actions prolong sensory blockade, enhance analgesia, and reduce systemic analgesic requirements [33-37].

Pharmacodynamic differences between clonidine and dexmedetomidine

Clonidine is a partial α2-adrenergic agonist with a receptor selectivity ratio of approximately 200:1 (α2:α1). While it is effective, its sedative and analgesic effects are less pronounced compared to those of dexmedetomidine [54]. This makes clonidine a useful option in certain clinical scenarios but with less potency in both sedation and pain relief [55]. On the other hand, dexmedetomidine is a highly selective α2-adrenergic agonist, with an α2:α1 selectivity ratio of nearly 1600:1. This enhanced selectivity confers significantly stronger analgesic and sedative properties, while also producing fewer α1-mediated side effects, such as hypertension, compared to clonidine [56]. Due to its higher receptor affinity, dexmedetomidine typically offers longer-lasting analgesia and deeper sedation at comparable doses.

The difference in selectivity between clonidine and dexmedetomidine has important clinical implications. Dexmedetomidine tends to produce more prolonged analgesia and deeper sedation, making it more suitable for situations requiring intense or extended postoperative pain management [39]. Conversely, clonidine may be preferred when a more balanced hemodynamic profile is necessary, particularly in patients who may be more sensitive to cardiovascular effects [57]. Table 3 further outlines the mechanistic and pharmacological distinctions between the two agents, highlighting how differences in receptor selectivity and site-specific actions can influence clinical outcomes.

**Table 3:** Mechanistic comparison of clonidine and dexmedetomidine as adjuvants.

Parameter	Clonidine	Dexmedetomidine	References
Receptor selectivity (α2:α1)	~200:1	~1600:1	[33,38]
Spinal action	Inhibits substance P release, prolongs sensory block	More potent inhibition of nociceptive transmission	[35,39]
Supraspinal action	Sedation via locus coeruleus suppression	Deeper sedation and anxiolysis	[36,39]
Peripheral action	Mild inhibition of norepinephrine release	Stronger membrane-stabilizing effect	[37,38]
Clinical impact	Balanced analgesia, modest sedation	Longer analgesia, deeper sedation	[38,39]

**Figure 1:**
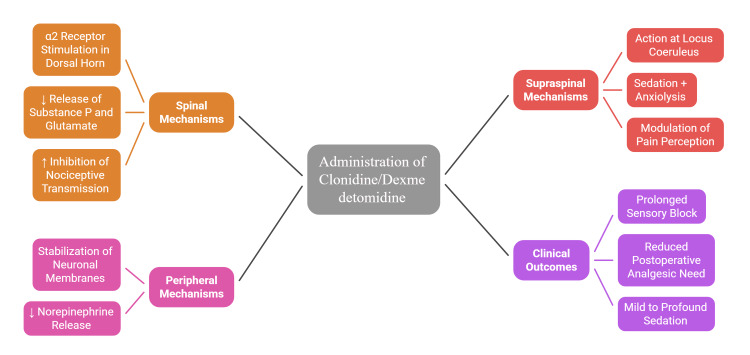
Mechanisms of action of α2-adrenergic agonists in caudal analgesia. Image credit: Khushboo Bairwa.

The mechanisms of action of α2-adrenergic agonists can be better understood through a stepwise representation (Figure 1), highlighting how clonidine and dexmedetomidine modulate nociception at spinal, supraspinal, and peripheral levels.

Clonidine as an adjuvant

Pharmacology of Clonidine

Clonidine is a partial α2-adrenergic agonist with a receptor selectivity ratio of ~200:1. It prolongs caudal analgesia primarily through spinal inhibition of nociceptive transmission and mild supraspinal sedation [40-42]. With a half-life of six to 12 hours and moderate lipid solubility, clonidine offers a sustained effect in single-shot caudal blocks. Recommended doses range from 1 to 2 µg/kg [43-45].

Clinical Evidence

Several clinical trials and reviews have demonstrated the benefits of clonidine as an adjuvant in pediatric caudal blocks.

Efficacy in prolonging analgesia: Randomized controlled trials have consistently shown that adding clonidine (1-2 µg/kg) to caudal local anesthetics such as bupivacaine or ropivacaine significantly prolongs analgesia duration compared to local anesthetics alone [46-48]. In particular, children undergoing infraumbilical or orthopedic surgeries experienced extended postoperative pain relief and reduced need for rescue analgesics [58].

Comparative efficacy: Studies comparing clonidine with other adjuvants (e.g., dexamethasone and nalbuphine) suggest clonidine offers superior prolongation of sensory block without excessive motor block, though it is less potent than dexmedetomidine in terms of analgesia duration [42,43].

Hemodynamic stability: In most pediatric trials, clonidine has been associated with stable intraoperative hemodynamics. Mild bradycardia and hypotension have been reported, but clinically significant cardiovascular depression is rare when appropriate dosing is observed [44]. Meta-analyses also reinforce clonidine’s role as a safe and effective caudal adjuvant, though heterogeneity in dosing and methodology across studies limits definitive recommendations [38-40].

Adverse Effects

Clonidine is generally well tolerated, but certain adverse effects should be carefully considered. Cardiovascular effects, such as hypotension and bradycardia, may occur due to reduced sympathetic outflow. These effects are typically dose-dependent and self-limiting, resolving once the drug is metabolized. Clonidine also produces mild to moderate sedation, which can be beneficial in the perioperative period [45]. However, this sedation may delay recovery in certain clinical settings, particularly in outpatient procedures where quick discharge is required. Other adverse effects reported with clonidine include dry mouth, urinary retention, and, although rarely in pediatric patients, prolonged motor block [46,59].

Despite these potential side effects, the overall risk-benefit profile of clonidine as a caudal adjuvant remains favorable, particularly when dosing is carefully titrated. Most pediatric studies recommend a dose range of 1-2 µg/kg, with higher doses increasing the risk of excessive sedation and hemodynamic compromise without providing additional benefits [59].

Dexmedetomidine as an adjuvant

Pharmacology of Dexmedetomidine

Dexmedetomidine is a highly selective α2-adrenergic agonist, with an α2:α1 selectivity ratio of approximately 1600:1, making it significantly more potent than clonidine. It enhances analgesia by strongly suppressing nociceptive transmission at the spinal cord level and provides deeper sedation by modulating the locus coeruleus [58]. Despite its relatively short terminal half-life of about two hours, dexmedetomidine's high receptor affinity ensures prolonged clinical effects, which are beneficial for postoperative pain management. Pediatric studies typically support dosing in the range of 1-2 µg/kg [48-51].

Clinical Evidence

Extensive clinical research consistently supports the efficacy of dexmedetomidine as a caudal adjuvant in pediatric patients. Numerous randomized controlled trials have demonstrated that when dexmedetomidine (at doses of 1-2 µg/kg) is added to bupivacaine or ropivacaine in caudal blocks, the duration of postoperative analgesia is significantly prolonged compared to when local anesthetics are used alone [52-54]. In some studies, analgesia has been extended beyond 12 hours, in contrast to the typical four to six hours provided by local anesthetics alone [55]. In addition to prolonging analgesia, dexmedetomidine improves block quality [53]. It enhances block density, reduces breakthrough pain episodes, and decreases the need for rescue analgesia in the immediate postoperative period [54]. Regarding sedation and recovery, children receiving dexmedetomidine often experience mild sedation during recovery, which aids in smoother emergence from anesthesia and reduces postoperative agitation, particularly after painful procedures. However, careful postoperative monitoring is essential, as excessive sedation could delay discharge from the recovery room [52,55]. Comparing dexmedetomidine with clonidine, head-to-head trials reveal that dexmedetomidine produces a longer duration of analgesia and deeper sedation when used as an adjuvant to caudal local anesthetics [52,55]. Although clonidine remains effective, dexmedetomidine is superior in extending analgesic benefits, which has led to its increasing clinical adoption in pediatric anesthesia.

Adverse Effects

Despite its numerous advantages, dexmedetomidine is not without adverse effects. The most commonly reported concerns are cardiovascular, including bradycardia and hypotension. These effects are due to enhanced vagal activity and reduced sympathetic outflow, and they are generally dose-related and reversible with appropriate supportive management [52,53]. Excessive sedation, while often beneficial for smoother recovery, can also delay discharge from the recovery room and complicate postoperative monitoring. Other side effects, although less frequent than those seen with opioids, include nausea, vomiting, and delayed motor recovery [54]. The optimal dosing range of 1-2 µg/kg appears to strike a balance between efficacy and safety. Higher doses carry the risk of excessive hemodynamic instability and sedation without offering proportional increases in analgesic benefit [55,56].

Comparative analysis: clonidine vs. dexmedetomidine

**Figure 2:**
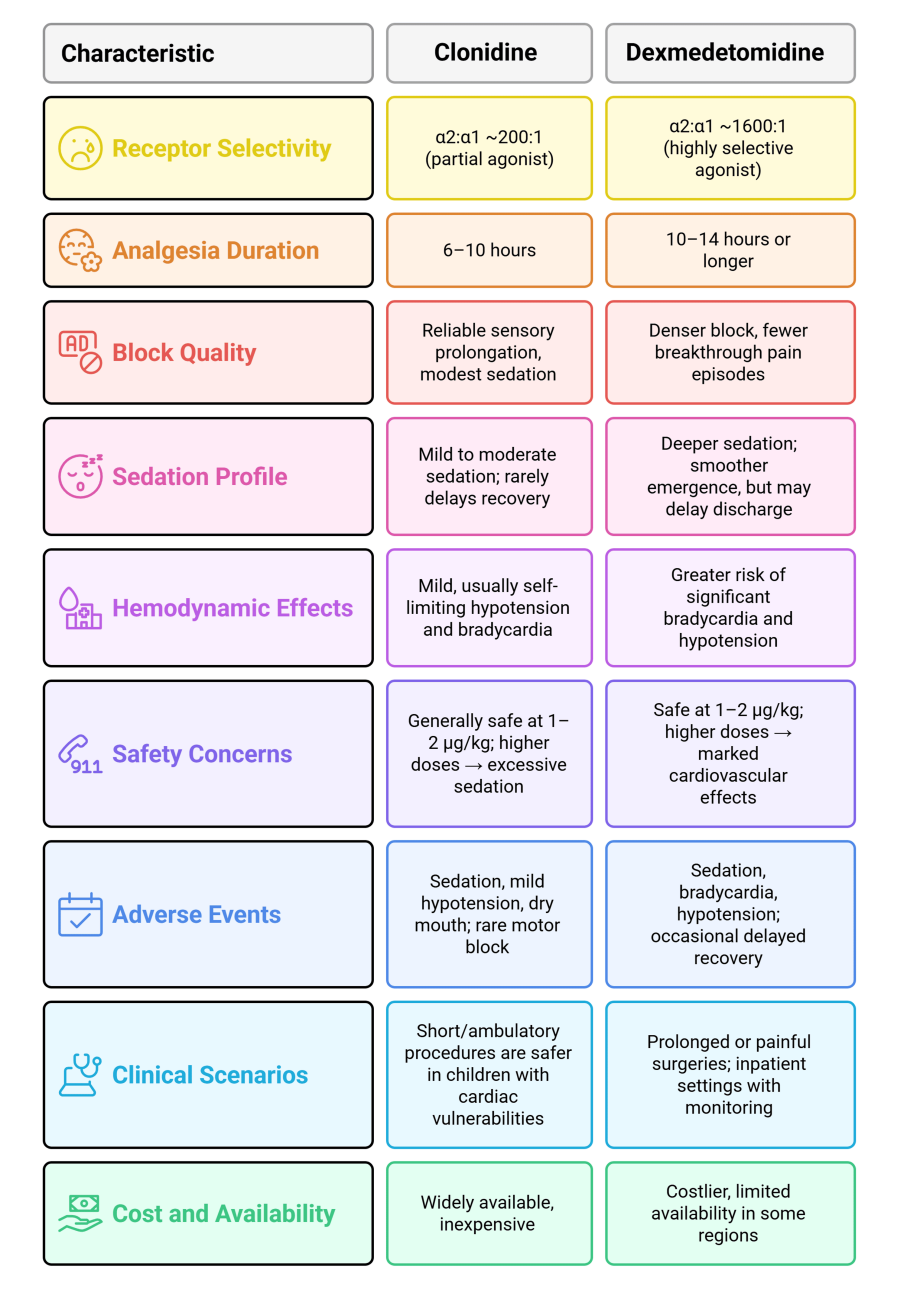
Comparative overview of clonidine vs. dexmedetomidine. Image credit: Created by the authors.

A comparative overview of clonidine and dexmedetomidine is shown in Figure 2, which summarizes their key differences, clinical applications, and limitations. Dexmedetomidine generally produces longer analgesia and deeper sedation at comparable doses, making it the preferred choice for surgeries requiring extended postoperative pain relief. Clonidine, while effective, may offer a more balanced hemodynamic profile, making it a safer option for patients with cardiovascular vulnerabilities or for shorter, ambulatory procedures [57].

Onset and Duration of Analgesia

Clonidine, when added to caudal local anesthetics, extends analgesia from three to six hours (with local anesthetic alone) to approximately six to 10 hours. Dexmedetomidine, by contrast, frequently prolongs analgesia beyond 12 hours, with some studies reporting durations extending to 14 hours [17,41,47,55]. The difference is largely attributed to dexmedetomidine’s higher α2:α1 selectivity, resulting in stronger suppression of nociceptive transmission [38,39]. This prolonged action with dexmedetomidine translates into fewer requirements for rescue analgesics in the postoperative period. Clonidine remains valuable, particularly in shorter procedures or when prolonged sedation is undesirable.

Sedation and Safety Profile

Both agents provide sedation via locus coeruleus suppression, though dexmedetomidine produces deeper and longer sedation compared to clonidine [9,11,16]. This may reduce emergence agitation but can delay discharge in ambulatory cases. Clonidine, on the other hand, produces milder sedation and more stable hemodynamics, making it preferable in children with cardiac vulnerabilities. Both drugs are safe within the 1-2 µg/kg range, though dexmedetomidine carries a higher risk of bradycardia and hypotension [59].

Cost and Availability Considerations

Clonidine is generally less expensive and more widely available than dexmedetomidine. In resource-limited settings, clonidine remains the practical choice. Dexmedetomidine, though costlier, may be justified in high-risk patients or surgeries where prolonged analgesia and sedation are desirable [6,51].

Clinical Preference in Pediatric Scenarios

Short ambulatory procedures: For shorter, ambulatory procedures, clonidine is typically the preferred choice. Its mild sedative effects and balanced recovery profile make it an excellent option for quick recovery, especially when postoperative sedation is not desired. This makes clonidine ideal for cases where a rapid return to normal functioning is a priority [8,21].

Longer or painful surgeries: In contrast, dexmedetomidine is better suited for longer or more painful surgeries. Its superior analgesic and sedative effects make it particularly effective in infraumbilical or orthopedic procedures, where extended postoperative comfort is essential. Dexmedetomidine’s ability to provide deep sedation and prolonged analgesia ensures that children undergoing such surgeries experience a more comfortable recovery [28,55].

High-risk patients: Clonidine is often considered a safer choice for children with cardiovascular vulnerabilities, such as those with pre-existing heart conditions. This is because clonidine generally has milder cardiovascular effects compared to dexmedetomidine, whose stronger sedative and bradycardic properties require careful monitoring, especially in high-risk patients [6,51].

A summary of clonidine and dexmedetomidine as caudal adjuvants, consolidating their pharmacological properties, clinical effects, and practical applications in pediatric anesthesia, is presented in Table 4. In resource-limited settings, clonidine's lower cost and broader availability make it the more practical choice, particularly where dexmedetomidine may be unavailable or financially prohibitive.

**Table 4:** Comparative profile of clonidine and dexmedetomidine as caudal adjuvants in children.

Parameter	Clonidine	Dexmedetomidine	References
Receptor selectivity (α2: α1)	~200:1 (partial agonist)	~1600:1 (highly selective agonist)	[33,38,39]
Analgesia duration (with local anesthetics)	6–10 hours	10–14 hours or longer	[17,41,47,55]
Block quality	Reliable sensory prolongation, modest sedation	Denser block, fewer breakthrough pain episodes	[38,39,52-55]
Sedation profile	Mild to moderate sedation; rarely delays recovery	Deeper sedation; smoother emergence, but may delay discharge	[16,18,19,39,54]
Hemodynamic effects	Mild, usually self-limiting hypotension and bradycardia	Greater risk of significant bradycardia and hypotension	[9,11,49,52]
Safety concerns	Generally safe at 1–2 µg/kg; higher doses → excessive sedation	Safe at 1–2 µg/kg; higher doses → marked cardiovascular effects	[45-47,53,56]
Adverse events	Sedation, mild hypotension, dry mouth; rare motor block	Sedation, bradycardia, hypotension; occasional delayed recovery	[23,29,37,52-54]
Clinical scenarios	Short/ambulatory procedures are safer in children with cardiac vulnerabilities	Prolonged or painful surgeries; inpatient settings with monitoring	[8,21,28,55]
Cost and availability	Widely available, inexpensive	Costlier, limited availability in some regions	[6,51]
Overall, role	Balanced efficacy and safety; suitable for most pediatric surgeries	Superior efficacy and sedation, but requires closer monitoring	[38,39,52-55]

Various potential adjuvants

Opioids such as morphine and fentanyl prolong caudal analgesia but are limited by risks of respiratory depression, nausea, vomiting, and pruritus, especially in children [13,55]. Ketamine provides N-methyl-D-aspartate (NMDA) receptor-mediated analgesia but raises concerns about neurotoxicity and inconsistent efficacy [13,45]. Neostigmine enhances spinal cholinergic transmission but is frequently associated with nausea and urinary retention [42,44]. Steroids like dexamethasone show promise but lack sufficient pediatric safety data [38]. Compared with these agents, clonidine and dexmedetomidine remain preferred due to their consistent efficacy, dual analgesic-sedative properties, and safer profiles [2,32].

Practical considerations

Optimal Dosing Strategies

The effectiveness and safety of α2-agonists in pediatric caudal blocks are closely linked to their dosing. For clonidine, the commonly recommended dose is 1-2 µg/kg when combined with local anesthetics. Doses below 1 µg/kg may not provide sufficient prolongation of analgesia, while doses above 2 µg/kg are associated with excessive sedation, bradycardia, and hypotension. Similarly, dexmedetomidine is typically dosed within the range of 1-2 µg/kg. At these doses, it effectively prolongs analgesia for up to 12-14 hours [60]. While higher doses of dexmedetomidine may extend analgesia further, they also significantly increase the risk of cardiovascular instability [17,21,24,31]. Therefore, careful titration of the dose is essential, particularly in infants and neonates, who are more sensitive to sedatives and may require closer monitoring to prevent adverse effects.

Combination With Local Anesthetics

The choice of local anesthetic plays a critical role in the overall efficacy of caudal blocks. Bupivacaine has long been used due to its effective sensory blockade but carries the risk of cardiotoxicity at high doses, which limits its use in certain pediatric cases [47]. Ropivacaine, a safer alternative, offers a similar duration and is often preferred in pediatric anesthesia due to its favorable safety profile [61]. Levobupivacaine, an enantiomer of bupivacaine, provides equivalent efficacy but with a reduced risk of cardiotoxicity. Studies have shown that adding clonidine or dexmedetomidine to any of these local anesthetics significantly prolongs analgesia without prolonging motor block, making them suitable for infraumbilical and lower limb surgeries [52-55].

Patient Selection and Contraindications

Not all pediatric patients are ideal candidates for α2-agonist adjuvants, and individual health conditions must be carefully considered. Healthy children undergoing infraumbilical or orthopedic surgeries are particularly suitable for α2-agonists like clonidine and dexmedetomidine, as these agents provide prolonged postoperative pain relief, particularly for surgeries requiring extended analgesia [22,34,38].

However, caution is required when administering dexmedetomidine to children with pre-existing cardiac conditions, such as conduction abnormalities or congenital heart disease, as these conditions increase the risk of bradycardia and hypotension, which are potential side effects of α2-agonists, particularly in more sensitive patients [60]. α2-agonists should also be avoided in children with significant hemodynamic instability or known hypersensitivity to these medications. Additionally, children with severe neurologic impairment are not suitable candidates, as the sedative effects of these agents could exacerbate neurological complications and make monitoring more difficult [22,34,38].

Perioperative Monitoring and Safety Precautions

Given the dose-dependent cardiovascular and sedative effects of α2-agonists, meticulous perioperative monitoring is crucial to ensure patient safety and effective management.

Intraoperative monitoring: Continuous monitoring is essential during caudal block administration when using clonidine or dexmedetomidine. This includes the use of continuous ECG to track heart rhythm, non-invasive blood pressure to assess hemodynamic stability, and pulse oximetry to monitor oxygen saturation [35]. These measures help detect any early signs of adverse effects, such as bradycardia or hypotension, ensuring prompt intervention if necessary [36].

Postoperative monitoring: After the procedure, children who have received clonidine or dexmedetomidine should be closely observed for signs of bradycardia, hypotension, and the depth of sedation [61]. While most side effects tend to be mild and self-limiting, interventions such as fluid boluses or the administration of atropine may be required in cases where the side effects are more pronounced [40].

Recovery considerations: Dexmedetomidine’s stronger sedative effect may delay recovery room discharge, making it less ideal for ambulatory surgeries where quick recovery is a priority. In such cases, clonidine is often preferred due to its milder sedation and more stable hemodynamics, ensuring a smoother and quicker recovery process [27,33,52].

Clinical Application and Balancing Risks

The decision to use either clonidine or dexmedetomidine as adjuvants in pediatric caudal anesthesia involves carefully weighing the analgesic benefits against the potential risks. Clonidine is known for its ability to moderately prolong analgesia while maintaining a stable safety profile [57]. This makes it a dependable option for most pediatric cases, particularly in outpatient procedures where mild sedation and cardiovascular stability are critical.

Dexmedetomidine, on the other hand, offers superior analgesia and deeper sedation. However, its use requires closer monitoring due to its potential cardiovascular effects, particularly bradycardia and hypotension. As such, dexmedetomidine may be more appropriate in inpatient settings, where extended monitoring is available, especially for surgeries that demand more intense postoperative pain management [58]. Clonidine’s milder cardiovascular effects make it a safer choice for children with congenital or acquired heart conditions. In these cases, dexmedetomidine’s more pronounced bradycardic effects may pose a higher risk, highlighting the importance of selecting the right adjuvant based on the patient’s health status and the surgical setting [35,36,40].

Future directions

Need for Large Multicenter Randomized Controlled Trials

Despite the promising results from existing studies on clonidine and dexmedetomidine, the evidence remains limited due to small sample sizes, heterogeneous methodologies, and the predominance of single-center designs. While meta-analyses have demonstrated their efficacy, definitive conclusions regarding the safety and effectiveness of these α2-agonists in pediatric caudal anesthesia can only be made through large, multicenter randomized controlled trials (RCTs). These trials would be instrumental in validating the outcomes across diverse pediatric populations and various surgical settings [1,15,32]. Furthermore, such studies should standardize dosing regimens, outcome measures, and monitoring protocols to enhance the reliability and applicability of their findings.

Long-Term Neurodevelopmental Safety

The potential neurotoxic effects of anesthetic and sedative drugs on the developing brain have raised concerns, particularly regarding their use in pediatric anesthesia. Preclinical studies suggest that both clonidine and dexmedetomidine may possess neuroprotective properties, which could offer benefits in minimizing neurodevelopmental risks. However, robust long-term data in pediatric populations are currently lacking [36,48]. Given that these α2-agonists act on central adrenergic pathways, well-designed longitudinal studies are critical to determine whether these drugs pose any adverse effects on neurocognitive development in children [62].

Integration Into Multimodal Pediatric Analgesia

Looking ahead, the integration of clonidine and dexmedetomidine into multimodal analgesia strategies is likely to become more prevalent. This approach combines these α2-agonists with systemic non-opioid agents, such as acetaminophen and NSAIDs, as well as regional techniques, with the aim of reducing opioid use, shortening recovery times, and improving overall perioperative outcomes [40,45]. Clinical trials should focus on identifying the optimal combinations of adjuvants and systemic agents, ensuring that the benefits are maximized while minimizing potential risks.

Personalized Analgesic Strategies

The concept of precision anesthesia, which tailors analgesic regimens to individual patient characteristics, is gaining increasing traction. This approach takes into account factors such as age, weight, genetic profile, and existing comorbidities. For example, clonidine may be a more suitable option for infants with cardiovascular vulnerabilities, while dexmedetomidine may be preferred in older children undergoing long orthopedic surgeries [40,62]. Future research should explore biomarkers and pharmacogenomics to predict patient responses to these drugs and identify those most at risk for adverse events.

Potential for Novel Delivery Methods

While single-shot caudal administration remains the standard practice, there is growing interest in exploring alternative delivery methods to optimize the use of clonidine and dexmedetomidine in pediatric anesthesia. One such approach is continuous caudal infusion, which could allow for lower cumulative doses of α2-agonists while maintaining prolonged analgesia. Additionally, novel formulations, such as liposomal preparations of local anesthetics combined with clonidine or dexmedetomidine, may provide ultra-long-acting analgesia. Expanding the use of these agents into peripheral nerve blocks and neuraxial techniques may also broaden their clinical applications, offering further opportunities for pain management in pediatric anesthesia [40,45].

Bridging Evidence and Clinical Practice

Despite strong clinical evidence, variability in practice remains due to differences in availability, cost, and institutional protocols. Dexmedetomidine, though effective, is often limited by its higher cost compared to clonidine [51]. Collaborative guidelines and consensus statements from pediatric anesthesiology societies are needed to establish evidence-based best practices.

Limitations of the review

This narrative review has certain limitations that warrant acknowledgment. As a non-systematic synthesis, it may be influenced by selection bias in the inclusion of studies. The number of randomized controlled trials available on pediatric caudal adjuvants remains limited, and there is considerable heterogeneity in dosing regimens, methodologies, and outcome measures across studies. Variations in patient age groups, surgical procedures, and anesthetic protocols further complicate direct comparison of results. Additionally, the inclusion of only English-language and recently published studies may introduce publication and language bias. These limitations underscore the need for large-scale, multicenter, and methodologically standardized trials to validate and extend the findings summarized in this review.

## Conclusions

Caudal epidural analgesia, while effective for pediatric infraumbilical and lower limb surgeries, is limited by its short duration when used with local anesthetics alone. The addition of α2-adrenergic agonists, such as clonidine and dexmedetomidine, significantly extends analgesia and enhances block quality. Clonidine provides a balanced profile with stable hemodynamics and is ideal for ambulatory procedures, while dexmedetomidine offers superior analgesia and deeper sedation, particularly for more extensive surgeries. The choice between these agents should be guided by the surgical context, patient factors, and recovery needs. Both drugs, when appropriately dosed, offer favorable safety profiles, making them valuable tools in pediatric caudal analgesia, though careful monitoring is essential to mitigate potential cardiovascular and sedative risks. Further research will help optimize their use in clinical practice.
